# Supplementary material for: Saccharomyces boulardii (CNCM I-745) improves intestinal damage in sepsis by remodeling intestinal flora structure
Source: Front Cell Infect Microbiol. 2025 Jul 31;15:1602792. doi: 10.3389/fcimb.2025.1602792 (PMC12350329; doi:10.3389/fcimb.2025.1602792)
Supplement: Supplementary file 1 [file DataSheet1.zip › summary/summary/7_Differential_analysis/1_diff_abundance/6_Genus/Group.BW4_vs_BW0/Group.BW4_vs_BW0_diff_barplot.pdf]

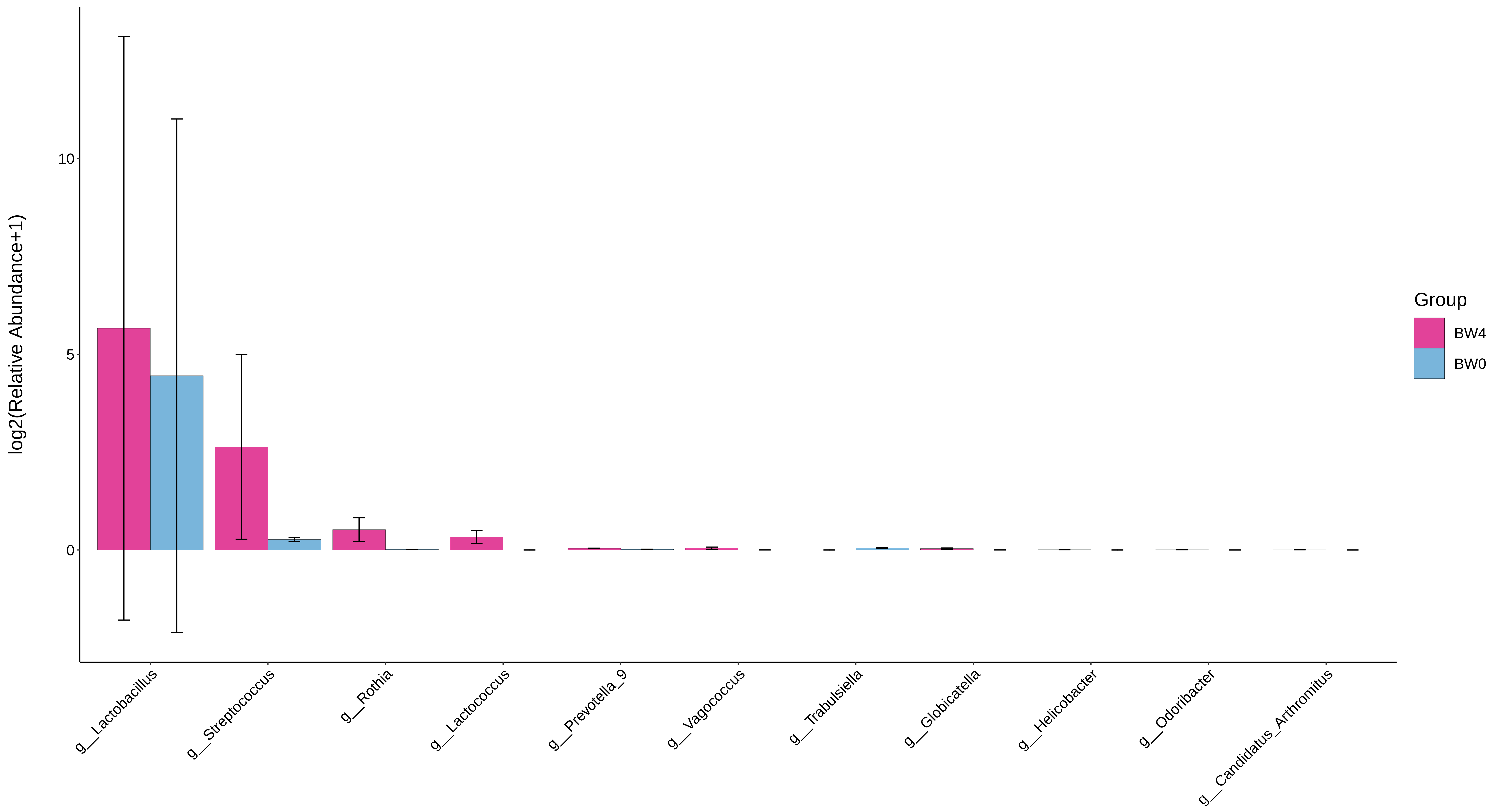

log2(Relative Abundance+1)
10
5
0
g__Lactobacillus
g__Streptococcus
g__Rothia
g__Lactococcus
g__Prevotella_9
g__Vagococcus
g__Trabulsiella
g__Globicatella
g__Helicobacter
g__Odoribacter
g__Candidatus_Arthromitus
Group
BW4
BW0
